# Functional high-intensity exercise is more effective in acutely increasing working memory than aerobic walking: an exploratory randomized, controlled trial

**DOI:** 10.1038/s41598-020-69139-z

**Published:** 2020-07-23

**Authors:** Jan Wilke

**Affiliations:** Department of Sports Medicine, Goethe University Frankfurt am Main, Ginnheimer Landstraße 39, 60487 Frankfurt am Main, Germany

**Keywords:** Cognitive neuroscience, Health care

## Abstract

Aerobic and resistance exercise acutely increase cognitive performance (CP). High-intensity functional training (HIFT) combines the characteristics of both regimes but its effect on CP is unclear. Thirty-five healthy individuals (26.7 ± 3.6 years, 18 females) were randomly allocated to three groups. The first (HIFT) performed a functional whole-body workout at maximal effort and in circuit format, while a second walked at 60% of the heart rate reserve (WALK). The third group remained physically inactive reading a book (CON). Before and after the 15-min intervention period, CP was assessed with the Stroop Test, Trail Making Test and Digit Span Test. Repeated-measures ANOVAs and post-hoc 95% confidence intervals (95% CI) were used to detect time/group differences. A significant group*time interaction was found for the backwards condition of the Digit Span Test (p = 0.04) and according to the 95% CI, HIFT was superior to WALK and CON. Analysis of the sum score of the Digit Span Test and the incongruent condition of the Stroop Test, furthermore, revealed main effects for time (p < 0.05) with HIFT being the only intervention improving CP. No differences were found for the Trail Making Test (p > 0.05). In conclusion, HIFT represents an appropriate method to acutely improve working memory, potentially being superior to moderate aerobic-type exercise.

## Introduction

Regular engagement in physical activity is linked to a variety of health benefits. Besides lowering the risk for cardiovascular diseases^1^, it can reduce all-cause mortality up to 33%^2,3^. In recent decades, it has also been shown that physical activity may prevent the development of neurodegenerative pathologies^4^. Although the mechanisms of this observation are yet to be elucidated, there is accumulating evidence revealing how particularly exercise, a planned and structured sub-set of physical activity performed at increased energy expenditure^5^, seems to induce both acute and chronic adaptations in the brain. Long-term endurance training of animals evoked angiogenesis, neurogenesis and enhanced synaptic plasticity^6–8^. Studies examining humans found expression of the brain-derived neurotrophic factor (BDNF) and increases in hippocampal volume occurred following several weeks of aerobic exercise^9^. After single endurance training bouts, substantial neurophysiological (e.g. increased delta, theta, alpha and beta detected by electromyography) and neurochemical (e.g. BDNF, insulin-like growth factor 1, dopamine, norepinephrine or serotonin) changes have been observed^10^.

In view of the strong and multifaceted response of the nervous system to exercise training, research has increasingly attempted to identify its impact on cognitive performance (CP), which can be subdivided in higher- (e.g. inhibitory control or working memory) and lower-order (e.g. attention or reaction time) functions. Available systematic reviews have mostly investigated the effects of aerobic-type exercise, detecting a positive effect on CP even when performed as a single training bout^11–13^. Interestingly, besides moderate continuous endurance exercise, high-intensity regimes may also have a positive impact. Although evidence is ambiguous in this regard^12–14^, Mandolesi et al.^15^ found the effects of related interventions to be superior to aerobic-type exercise in some populations. A recent meta-analysis, furthermore, concluded that resistance training improves CP in the short-term, being as effective as endurance exercise^16^. It may consequently be expected that a high-intensity workout combining elements of both endurance and resistance exercise regimes represents an intriguing method to enhance brain function.

High-intensity functional training (HIFT) is a popular fitness trend, which integrates cardiovascular and muscular effort by means of complex, partly loaded movement patterns with only minimal breaks in-between^17^. Related all-out workouts have been shown to trigger positive adaptations in endurance and strength capacities^18,19^. However, the effects of HIFT on CP are unclear. The present trial therefore aimed to elucidate the immediate impact of a single HIFT exercise bout on measures of brain function. It was hypothesized that HIFT would elicit greater improvements in CP than aerobic-type exercise and physical inactivity.

## Methods

### Ethical standards and study design

The study is part of the COINS (COgnition and INjury in Sports) network project. A three-armed, randomized, controlled trial, following the CONSORT (Consolidated Standards of Reporting Trials) guidelines was performed^20^. It was prospectively registered at the German Register of Clinical Trials (DRKS00017372, 12/09/2019) and conducted in accordance with the Declaration of Helsinki including its recent modification of Fortaleza (2013). Ethical approval was obtained from the local review board (Ethics committee of the Faculty of Psychology and Sports Sciences, Goethe University, Frankfurt) and each volunteer signed informed consent prior to study inclusion.

After screening for eligibility, enrolled participants were randomly allocated to three groups: (1) high-intensity functional circuit training (HIFT), (2) moderate-intensity walking exercise (WALK) or (3) physical inactivity control/reading (CON). Prior to and after the intervention, outcomes of CP were assessed. All participants visited the laboratory twice with a one-week interval between. While the first appointment served as a familiarization session regarding the cognitive tests and (in case of randomization in the corresponding group) the exercises of the HIFT workout, the actual experiment was performed on the second appointment. Randomization was performed using the software package “BiAS for Windows”, version 9.05 (Goethe-University Frankfurt, Germany).

### Participants

A sample of n = 35 exercise science undergraduate and graduate students (26.7 ± 3.6 years, 18 females) were recruited in October 2019 by means of personal contact and poster advertising at the university campus. Besides being healthy, they had to habitually engage in a minimum of five sporting hours per week. The most frequent sports performed were running, football (soccer), handball and basketball. Exclusion criteria encompassed (a) severe orthopaedic, cardiovascular, pulmonary, neurological, psychiatric or inflammatory rheumatic diseases, (b) pregnancy or nursing period, (c) analgesic intake during the trial or in the 48 h prior to study enrollment, (d) impairments in color vision, and (e) history of surgery or trauma in the lower extremity.

### Intervention

The intervention of the HIFT group was validated in a previous trial^18^. It consisted of 15 functional whole-body exercises performed in a circuit format with repetitive 20 s all-out training bouts and 10 s rest periods. With a total duration of 15 min, one workout thus had 30 exercise cycles. The selection of the exercises was based on two main goals: (a) the involvement of major muscle groups to increase absolute oxygen consumption and (b) the simulation of daily used fundamental movement patterns (e.g. Squat, Lunge, Push-Up). Prior to the workout, a short general warm-up (rope jumping) was performed. During the training, which was performed indoors, the participants were encouraged to attain maximum workload (rather by increasing repetitions per bout than by increasing weights) while maintaining high movement quality, which was continuously monitored by a specifically trained instructor holding a Bachelor’s degree in exercise science. If required, modifications of the exercises (e.g. Push-up on knees for some women and men with insufficient strength) were offered. To facilitate the achievement of maximal workout intensity, music (140–160 beats per minute) was played^17^.

Aerobic exercise, which has been mostly used as an exercise modality in previous studies, was chosen as an active comparison. The participants in the corresponding group (WALK) performed 15 min of treadmill walking at 60% of the individual heart rate reserve (HRR)^18^. HRR was determined by means of the Karvonen formula (resting heart rate + ((maximal heart rate – resting heart rate) x intensity). While resting heart rate was measured using a heart rate monitor, the maximal rate was estimated as 208 – 0.7 × age^21^. During the WALK intervention, maintaining the calculated individual heart rate values was ensured via continuous heart rate monitoring (Beurer PM80, Beurer GmbH, Ulm, Germany). To achieve maximal comparability to the HIFT intervention, the participants were also offered listening to music during walking. Preceding both interventions (HIFT and walking), all participants performed a short standardized and identical warm-up by walking on the spot for 60 s.

The third group functioned as a passive control condition. Here, participants were physically inactive. Seated on a chair, they were provided with a book on exercise physiology and instructed to read for 15 min (topics/pages of free choice). Prior to being provided with the book, the participants sat on the chair for 1 min in order to match the warm-up duration in the other groups. In all three conditions, a special focus was on ensuring comparable social attention. Necessary instructions were provided pre-intervention whereas only minimal factual feedback was given during exercise/reading.

### Outcomes

Before and after the intervention, markers of cognitive function were measured. To prevent learning effects, three strategies were used^22^. Firstly, on a separate day, all participants completed a familiarization session with three repetitions of each test. Secondly, prior to the actual assessments, one warm-up trial was performed. Finally, no identical tests forms (different color/ number orders) were applied. Testing order was randomized and the delay between the end of the experimental condition and the start of the post-measurements was standardized amounting to 30 s.

The *Stroop test* has three parts. In the first and second section capturing attention, the participants are asked to name the words written or colours displayed on a sheet as quickly as possible. The third section represents a measure of inhibition control. Words of colors are listed incongruently (e.g. “green” written in red or “blue written in yellow). Here, the participants needed to name the color of the word while ignoring the letters. For later analysis, the time needed to complete the task was recorded. The Stroop test has been demonstrated to display high reliability (ICC: 0.82) and internal consistency (Cronbach’s alpha: 0.93–0.97)^23^.

The *Trail Making test *(TMT) consists of two parts. In part A, the participants were required to connect linearly increasing numbers using a pen at maximal possible speed. In part B, successive numbers and letters (e.g., 1 to a to 2 to b) were to be linked alternatingly. Similar to the Stroop test, time needed for completion was recorded. The results of the test provide a measure of visual screening/attention (TMT-A) and cognitive flexibility/working memory (TMT-B). High reliability (ICC: 0.81–0.86) and construct validity of the TMT have been shown^24,25^.

In the *Digit Span* test, two conditions are performed. In the first, the participants need to memorize and repeat increasing amounts of numbers read to them. At the beginning, four numbers are to be recalled. In case of successful memorization, five numbers are named. For each step, two repetitions are performed and one or zero points are awarded depending on recall success. The test ends if both trials are failed. The second condition is identical to the first but the numbers have to be repeated in reversed order (e.g. 2, 4, 7, 9 becomes 9, 7, 4, 2). Both test parts and the composite score are linked to short-term and working memory^26^. The Digit Span test is reliable for repeated measurements (r = 0.73)^27^. Prior to starting outcome assessments, subjective arousal (Likert scale from ‘0—not activated’ to ‘6—highly activated’) and concentration (10 cm Visual Analogue Scale, 0 = not concentrated at all to 10 = highly concentrated) were assessed. Additionally, after the interventions, the participants stated their rate of perceived exertion (6–20 RPE scale^28^) as well as enjoyment of the intervention (Likert scale from ‘0—not fun at all’ to ‘6—most possible fun’).

### Data processing and statistics

For interval scaled data, means and standard deviations and for ordinal data, medians and minimums/maximums were computed. For interval data (e.g. time recorded in Stroop/TMT or points in Digit Span test), after checking the underlying assumptions of normal distribution of residuals and variance homogeneity, repeated measures ANOVAs (2 × 3) were performed to detect differences in time (pre-to-post intervention) and between groups. In case of significance of the omnibus test, 95% confidence intervals of the pre-post changes were constructed to identify the exact location of systematic pre-post/between-group differences. In all analyses, p values < 0.05 were considered to be significant. Calculations were made with “SPSS Statistics”, version 24 (IBM, SPSS Inc., Chicago, IL, USA) and “BiAS for Windows”, version 9.05 (Goethe-University Frankfurt, Germany).

## Results

The three groups were not different regarding age, sex, height, weight, arousal, concentration and cognitive baseline performance (p < 0.05; Table 1). All individuals completed the study and no drop-outs occurred.

**Table 1:** Characteristics and pre-intervention values in the three groups.

	Control	Walking	HIFT	p value
Age [yrs.]	27.3 ± 3.8	26.5 ± 4.4	26.4 ± 2.6	0.84
Height [cm]	172.4 ± 9.4	174.5 ± 10.9	175.5 ± 9.2	0.73
Weight [kg]	67.2 ± 9.6	67 ± 14.8	71.3 ± 10.5	0.61
Sex	8 ♂, 4 ♀	4 ♂, 7 ♀	5 ♂, 7 ♀	0.30
Arousal	4 ± 0.9	4.4 ± 0.7	3.8 ± 0.7	0.21
Concentration	3.8 ± 1.2	4.4 ± 1.2	3.5 ± 0.9	0.28
Stroop word (t)	25.3 ± 3.3	25.8 ± 4.4	26.3 ± 2.4	0.79
Stroop color (t)	37.8 ± 5.7	33.6 ± 5.1	34 ± 4.5	0.10
Stroop interference (t)	51.5 ± 11.4	52.2 ± 13.2	58.2 ± 7.7	0.28
Trail making test A (t)	28.2 ± 10.8	23.5 ± 8.9	20.8 ± 9.2	0.19
Trail making test B (t)	26.4 ± 13.6	25.6 ± 12.4	30.4 ± 13.4	0.64
Digit span forward (pts)	6.5 ± 2	8 ± 2.3	6.9 ± 2	0.23
Digit span backward (pts)	5.1 ± 2.2	5.3 ± 2.3	4.5 ± 1.7	0.65
Digit span score (pts)	11.6 ± 4	13.3 ± 4.4	11.3 ± 3.2	0.42

Table shows means and standard deviations for interval scaled data and medians including range for ordinal scaled data.

*Yrs.* years, *cm* centimeters, *kg* kilogram, *t* time in seconds, *pts* points.

The participants of the two exercise conditions found their activity more enjoyable than individuals randomized to CON (p < 0.001, d = 2.6). The highest values were recorded in HIFT (4.8 ± 0.7) although the difference to WALK (3.9 ± 0.8) did not reach statistical significance (p = 0.17). Perceived exertion was highest in HIFT (RPE: 16 ± 1) when compared to WALK (13.3 ± 1.6) and CON (6; p < 0.0001).

**Figure 1:**
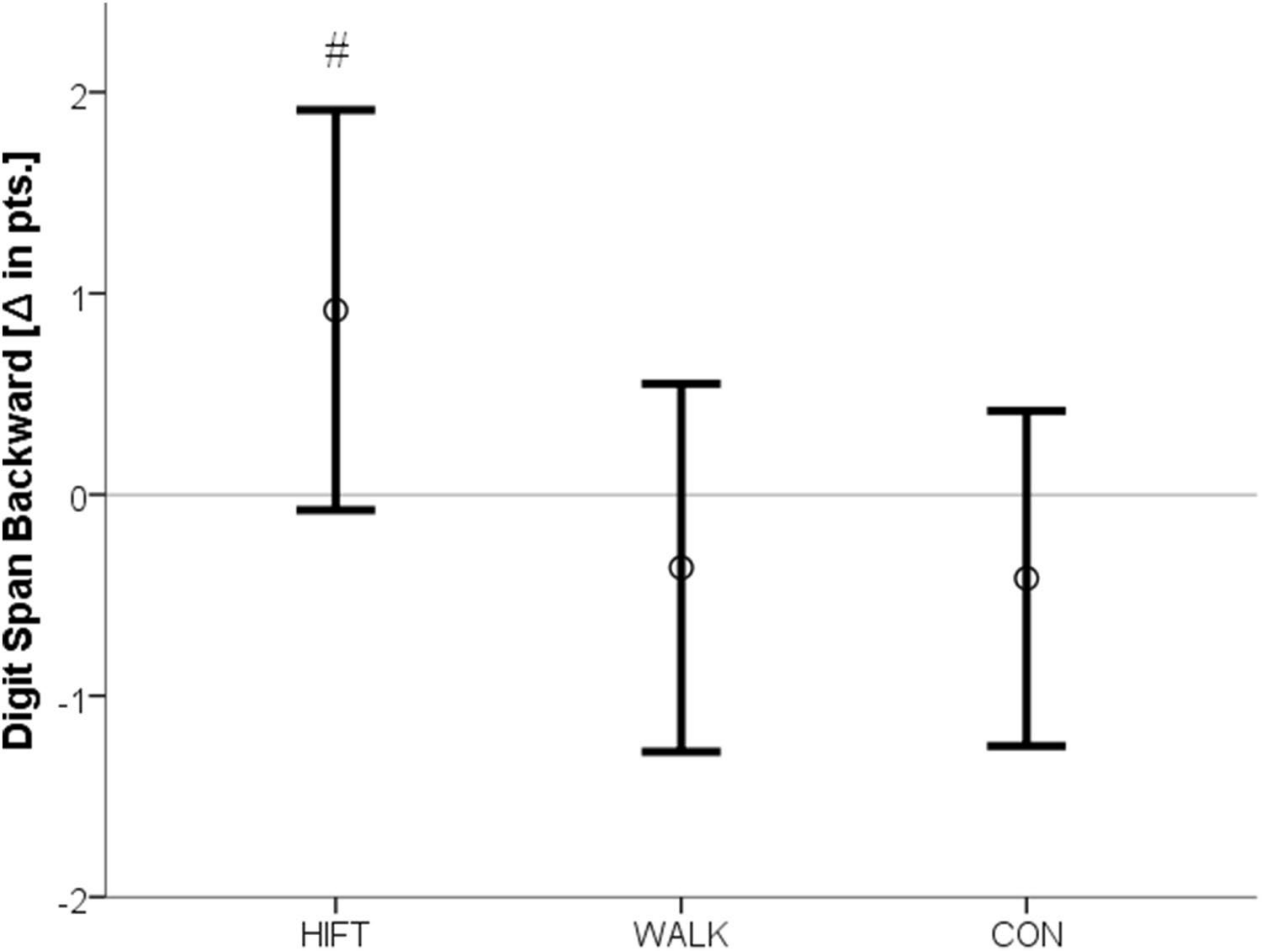
Pre-post differences in short-term/working memory (digit span backwards) are displayed as a function of activity type. Figure shows means and 95% confidence intervals. *Pts* points, ^#^significant group*time interaction.

**Figure 2:**
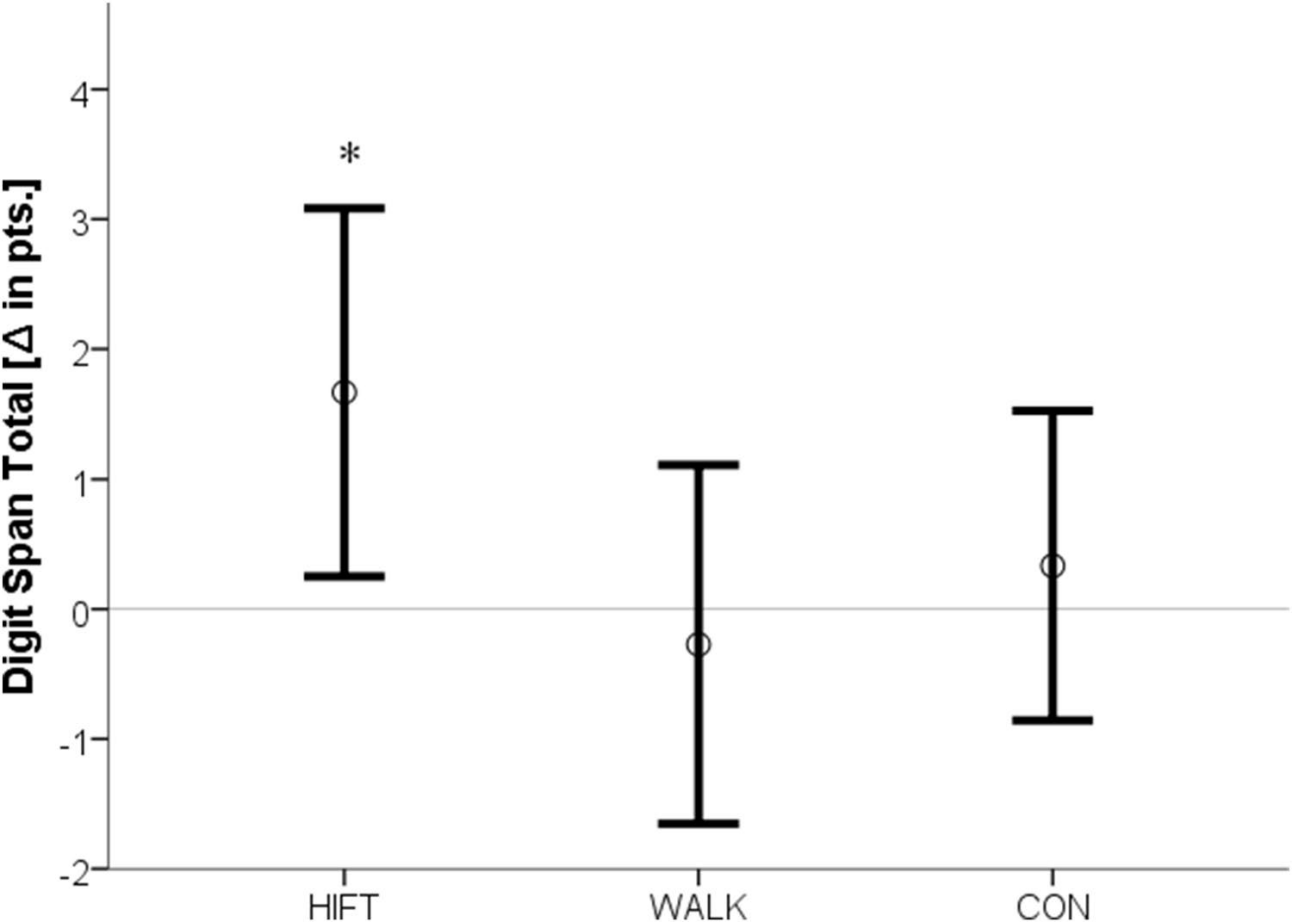
Pre-post differences in short-term/working memory composite rating (digit span total) as a function of activity type. Figure shows means and 95% confidence intervals. *Pts* points, *significant main effect for time.

**Figure 3:**
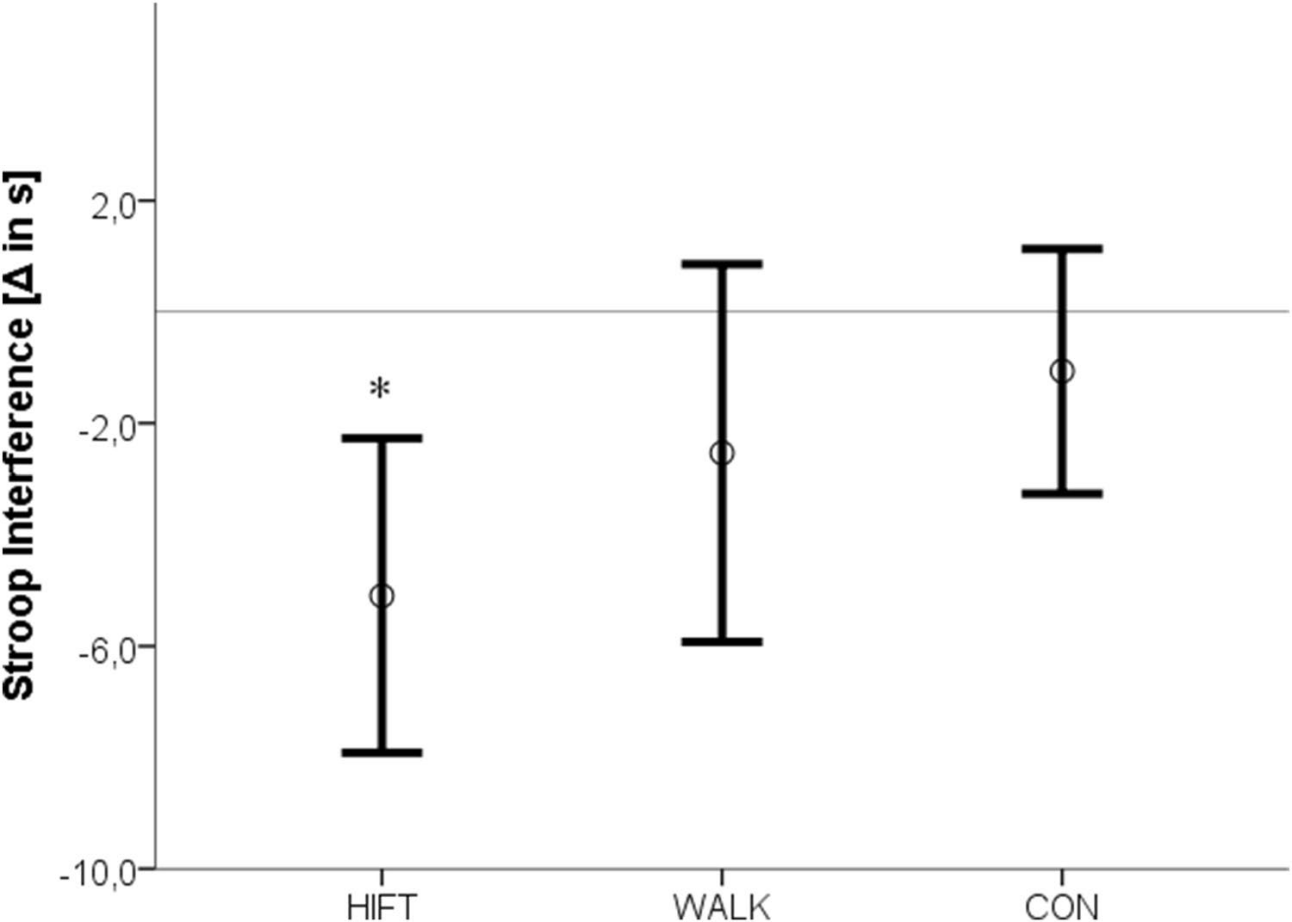
Pre-post differences in inhibitory control (stroop interference test) as a function of activity type. Figure shows means and 95% confidence intervals, *significant main effect for time.

The cognitive performance pre- to post-changes in the three groups are displayed in Table 2. A significant group × interaction [F(2,32) = 3.36, p = 0.047] was detected for the backwards condition of the Digit Span Test. Post hoc analysis of the confidence intervals revealed that HIFT increased short-term/working memory when compared to the other two conditions (Fig. 1). The tests for group × time interactions in the sum score of the Digit Span Test [F(2) = 2.74, p = 0.08] and the incongruent condition of the Stroop test [F(2) = 2.65, p = 0.09 eta^2^ = 0.14] approached but failed to achieve statistical significance. However, main effects for time [Stroop incongruent: F(1) = 15.56, p = 0.0001, Digit Span composite: F(1) = 3.94, p = 0.04] were found and, according to the confidence intervals, systematic increases were found only in the HIFT condition (Figs. 2, 3). No between-group or time differences occurred for the Trail Making Test.

**Table 2:** Pre-post differences in cognitive measures as a function of activity type.

	Control	Walking	HIFT	Time × group	Time
Stroop word (t)	0.2 ± 2.9	− 1.3 ± 2.6	− 0.7 ± 2.4	F(2) = 0.91, p = 0.41, eta^2^ = 0.05	F(1) = 1.69, p = 0.20
Stroop color (t)	− 0.4 ± 5.4	− 0.2 ± 2.9	− 3.2 ± 3.4	F(2) = 2.1, p = 0.15 eta^2^ = 0.11	F(1) = 3.22, p = 0.08
Stroop interference (t)	− 1.06 ± 5.7	− 2.5 ± 5	− 5.1 ± 4.4	F(2) = 2.65, p = 0.09 eta^2^ = 0.14	F(1) = 15.56, p = 0.0001*
Trail making test A (t)	0.3 ± 5.2	− 1 ± 6.9	− 5 ± 9.4	F(2) = 1.67, p = 0.20 eta^2^ = 0.09	F(1) = 2.31, p = 0.14
Trail making test B (t)	− 4.7 ± 9.6	0.9 ± 17.7	− 1.5 ± 12.3	F(2) = 0.85, p = 0.44 eta^2^ = 0.03	F(1) = 0.27, p = 0.61
Digit span forward (pts)	0.75 ± 1.7	0.1 ± 1.4	0.3 ± 1.8	F(2) = 0.49, p = 0.62 eta^2^ = 0.03	F(1) = 1.65, p = 0.21
Digit span backward (pts)	− 0.4 ± 1.3	− 0.4 ± 1.4	0.9 ± 1.6	F(2) = 3.36, p = 0.04*, eta^2^ = 0.17	F(1) = 0.04, p = 0.85
Digit span score (pts)	0.3 ± 1.9	− 0.3 ± 2.1	1.7 ± 2.2	F(2) = 2.74, p = 0.08 eta^2^ = 0.14	F(1) = 3.94, p = 0.04*

*t* time in seconds, *pts* points, *ES* effect size.

Asterisks mark statistical significance.

## Discussion

The results of the present study suggest that HIFT can acutely improve CP, namely short-term/working memory and inhibitory control. Verifying our hypothesis, the high-intensity workout combining elements of endurance and resistance training was slightly superior to the aerobic exercise condition. The mechanisms by which HIFT increases CP merit further elaboration. Enhanced cerebral perfusion has been observed following endurance exercise^29,30^. Resistance training leads to increases the serum levels of the stress hormone cortisol^31^ and higher circulating BDNF concentrations^32^. Possibly, these factors, acting in concert, may play a role in the genesis of the observed effects.

While the impact of HIFT on CP had not been investigated before, our finding that the aerobic condition did not affect CP is in contrast to previous trials. This is of interest because it had been repeatedly claimed that moderate to vigorous intensities would be effective in increasing CP. Based on their literature review, Brisswalter et al.^33^ assumed a “sweet spot” at levels between 40 and 80% of the maximal oxygen uptake. Chang et al.^12^ found a significant impact of very light to moderate exercise on various markers of CP but no influence of hard or very hard exercise. Regarding the latter, evidence seems conflicting. While there are reports of increased CP following high-intensity exercise in elderly persons^15^, Browne et al.^14^ did not make similar findings in trained athletes which may point towards a population specificity. Notwithstanding, their conclusion that multiple factors such as fitness levels and exercise mode influence the exercise-cognition relationship^14^ matches with our results. Possibly, our HIFT workout with its high contextual variation, non-prescribed heart rate and the required attentional demand on movement execution has substantially different effects than rather monotonous activities such as cycling or running. This hypothesis should particularly be tested because most theories regarding the effect of training intensity on CP are based on aerobic-type exercise^10,12,14,33^.

HIFT has experienced a recent surge in popularity in both recreational sports and rehabilitation settings^17^. However, evidence for its effectiveness has been anecdotal for a long time. A significant practical implication of the present study is that HIFT represents an alternative to the use of other conventional training methods not only in terms of motor function but in addition when aiming to acutely improve CP. Yet, HIFT could also be of interest for sedentary individuals. Large shares of the population do not meet current physical activity guidelines, which recommend engagement in different kinds of exercise including endurance, resistance and balance training^33^. Lacks of motivation and time are frequently reported as barriers to the engagement in physical activity. In a previous study, we have shown that HIFT creates stronger intrinsic motivation and higher exercise enjoyment than a moderate aerobic-type activity^18^. Because it, furthermore, can concurrently improve muscular and cardiovascular function^18,19^ as well as CP in the short-term, HIFT may represent an optimal method for the target group of individuals with limited motivation and time to exercise. Nonetheless, with regard to CP improvements, it has to be noted that the sustainability of the CP improvement is yet to be investigated. Whereas this study shows an immediate effect of HIFT, future studies should include additional follow-up assessments and investigate chronic CP changes following long-term training.

Some methodological shortcomings warrant consideration. Using a robust three-armed RCT design, our study questions the occasionally proposed hypothesis that higher exercise intensities are, per se, less effective in immediately improving CP^12^. However, although this study provides intriguing evidence for the effectiveness of HIFT, it did not include a comparison against an intensity-matched endurance or resistance training bout. It is, hence, not possible to draw definite conclusions regarding the exercise character. Another aspect relates to the sample size. Due to the exploratory nature of this trial, no sample size calculation was performed. With n = 35 participants, the trial, therefore, may have lacked power to detect further differences between the disposed interventions. This is supported by the inspection of the error bars: For instance, the confidence intervals of the Trail Making test showed a similar trend to the other outcomes (highest improvement after HIFT) but due to their broadness, there was a slight overlap between the bars. Consequently, and in view of the mostly large effect sizes, further research with larger samples may detect additional between-group differences of smaller size.

## Conclusion

HIFT is an effective method to acutely enhance CP, namely working memory, being slightly superior to aerobic walking exercise. This finding questions the often-held belief that moderate intensities are optimal to increasing brain function. Besides further elucidating the general importance of exercise intensity, upcoming research should therefore examine the impact of specific exercise characteristics such as contextual variation, attentional demand and self-determination.

## Data Availability

Data will be made available on request.
